# Validity and reliability of the persian version of the modified telephone interview for cognitive status among community-dwelling older adults in Iran

**DOI:** 10.1590/1980-5764-DN-2023-0020

**Published:** 2024-01-05

**Authors:** Fatemeh Ghonoodi, Farshad Sharifi, Hooman Shahsavari, Sahar Keyvanloo Shahrestanaki, Elham Navab

**Affiliations:** 1 Tehran University of Medical Sciences, School of Nursing and Midwifery, Department of Geriatric Nursing, Tehran, Iran.; 2 Tehran University of Medical Sciences, Elderly Health Research Center, Endocrinology and Metabolism Population Sciences Institute, Tehran, Iran.; 3 Tehran University of Medical Sciences, School of Nursing and Midwifery, Department of Medical-Surgical Nursing, Tehran, Iran.; 4 Iran University of Medical Sciences, School of Nursing and Midwifery, Tehran, Iran.; 5 Tehran University of Medical Sciences, School of Nursing and Midwifery, Department of Geriatric Nursing, Tehran, Iran.; 6 Tehran University of Medical Sciences, School of Nursing and Midwifery, Department of Critical Care and Geriatric Nursing, Tehran, Iran.

**Keywords:** Information Technology, Interviews as Topic, Modified Telephone Interview for Cognitive Status, Psychometrics, Cognitive Dysfunction, Aged, Sensitivity and Specificity, Tecnologia da Informação, Entrevistas como Assunto, Modified Telephone Interview for Cognitive Status, Psicometria, Disfunção Cognitiva, Idoso, Sensibilidade e Especificidade

## Abstract

Due to the increase in the population of the elderly, there is a growing trend in some diseases such as cognitive disorders (dementia) which are common in this era, and the diagnosis and treatment of this disease are still facing challenges. Therefore, early identification of cognitive disorders is of particular importance. In this regard, the use of any tool or cognitive tests may not be enough to diagnose dementia in the early stages and a special tool is needed.

**Objective:**

The validity and reliability of the Persian version of the Modified Telephone Interview for Cognitive Status (P-TICS-M) in older adults living in the Iranian community for a comprehensive screening of mild cognitive impairment and dementia was investigated.

**Methods:**

In the first phase, translation, re-translation, and word-taking were performed by using the face validity and content validity. In the second phase, a stratified convenient sampling with 150 participants aged ≥60 years was conducted based on cognitive status using the global deterioration scale in 2018. The external and internal reliability of the P-TICS-M using the interclass correlation coefficient and Cronbach's alpha coefﬁcient of total items of this tool were estimated.

**Results:**

The mean age of the participants was 68.6 (standard deviation±7.4) years. According to global deterioration scale, 87 (58.0%) had normal cognition, 40 (26.7%) had mild cognitive impairment, and 23 (15.3%) had dementia. The Spearman's correlation coefficient between P-TICS-M scores and Mini-Mental State Examination scale was 0.764. In exploratory factor analysis, seven domains were detected, which were compatible with those defined by the tool developer. The Cronbach's alpha of the P-TICS-M was 0.920. The absolute agreement between test-retest score was >0.90. The sensitivity of 92.2, 94.8, and 100%, and also the specificity of 79.4, 88.2, and 89.8% were calculated for detecting subjects with dementia, respectively. Furthermore, a mild cognitive impairment cutoff of >28 was determined.

**Conclusion:**

The development and validation of a P-TICS-M tool can be useful in identifying older adult people with cognitive impairment. Demographic characteristics (level of education, age) can also affect the cutoff point of this tool.

## RESUMO.

Por causa do aumento da população de idosos, há uma tendência crescente de algumas doenças, como os distúrbios cognitivos (demência), que são comuns nessa época, e o diagnóstico e tratamento dessa doença ainda enfrentam desafios. A identificação precoce de distúrbios cognitivos é de particular importância. Nesse sentido, a utilização de qualquer ferramenta ou testes cognitivos pode não ser suficiente para diagnosticar a demência nas fases iniciais e é necessária uma ferramenta especial.

**Objetivo:**

A validade e a confiabilidade da versão persa da Entrevista Telefônica para o Estado Cognitivo - Modificada (P-TICS-M) em idosos que vivem na comunidade iraniana para uma triagem abrangente de comprometimento cognitivo leve e demência foram investigadas.

**Métodos:**

Primeira fase, tradução, retradução e tomada de palavras utilizando validade de face e validade de conteúdo. Na segunda fase, foi conduzida uma amostragem estratificada por conveniência com 150 participantes com idade ≥60 anos baseada em estado cognitivo por meio da escala de deterioração global em 2018. Estimaram-se a confiabilidade externa e interna do P-TICS-M por meio do coeficiente de correlação interclasses e o coeficiente alfa de Cronbach do total de itens deste instrumento.

**Resultados:**

A média de idade dos participantes foi de 68,6 (desvio padrão±7,4) anos. De acordo com a escala de deterioração global, 87 (58,0%) apresentavam cognição normal, 40 (26,7%) apresentavam comprometimento cognitivo leve e 23 (15,3%) apresentavam demência. O coeficiente de correlação de Spearman entre os escores do P-TICS-M e a escala do Mini-Exame do Estado Mental foi de 0,764. Na análise fatorial exploratória, detectaram-se sete domínios, os quais eram compatíveis com aqueles definidos pelo desenvolvedor da ferramenta. O alfa de Cronbach do P-TICS-M foi de 0,920. A concordância absoluta entre o escore teste-reteste foi >0,90. Calculou-se, respectivamente, sensibilidade de 92,2, 94,8 e 100%, e também especificidade de 79,4, 88,2 e 89,8% para a detecção de indivíduos com demência. Além disso, determinou-se um ponto de corte do comprometimento cognitivo leve >28.

**Conclusão:**

O desenvolvimento e validação de uma ferramenta P-TICS-M pode ser útil na identificação de idosos com comprometimento cognitivo. As características demográficas (escolaridade, idade) também podem afetar o ponto de corte dessa ferramenta.

## INTRODUCTION

The number of people with dementia is indeed increasing worldwide. The global dementia epidemic is a consequence of an aging population. It is estimated that every three seconds, someone in the world develops dementia. As the global population ages, the number of people living with dementia is expected to triple by 2050^1^. The highest prevalence of dementia is in Asia and is projected to increase. In Iran, the prevalence of dementia is 8.1% and increases with age^2^.

Dementia is a major public health concern, and the World Health Organization (WHO) has recognized the need for a global action plan to address this issue. The WHO released a global action plan on the public health response to dementia, providing guidelines and recommendations for countries to improve their response to the disease. One of the goals outlined in the action plan is that by 2025, at least half of the countries should have measures in place to recognize and diagnose at least half of the estimated number of people with dementia^3^.

Early diagnosis of cognitive impairment and intervention play a crucial role in altering the course of the disease and slowing down its progression^4^. Early detection of cognitive impairments like dementia offers numerous advantages, including the preservation of independence, improved quality of life^5^, better planning, access to support services, and potential cost savings. It underscores the importance of regular cognitive screenings and seeking medical attention if any concerns arise about cognitive function^6^.

Numerous screening instruments have been developed in recent years to assess cognitive status. However, many of these instruments face challenges in their application and may not be suitable for certain population groups, particularly those who are illiterate or have limited education^7^. Particularly, in developing countries such as Iran, where more than 50% of the older population are illiterate or near illiterate^6,8,9^, many of these tools are not applicable^6^.

From an epidemiological perspective, it is important to have instruments that are easily administered, consume less time, and can screen a large sample of older individuals at a lower cost^7^. Telephone-based tools for cognitive impairment screening are very interesting because they are very convenient for application^10^. In other words, there is no need to recall older adults for face-to-face interviewing, which is very difficult and resource-consuming^5^.

Telephone interview for cognitive status (TICS) is a global cognitive function assessment tool utilized both over the telephone and face-to-face method. The TICS indicates high sensitivity (94%) and specificity (100%) in recognizing participants with Alzheimer's disease (AD) among normal ones. In addition, this tool can be used in people with visual or motor impairments. The domains measured by the TICS are orientation, concentration, short-term memory, language, praxis, and mathematical skills^11^.

Modified telephone interview for cognitive status (TICS-M) was developed according to TICS in 1989 and had sufficient psychometric properties in many languages and cultures^12,13^. The validity and reliability of TICS-M in Persian language (P-TICS-M) have not been evaluated so far. This study was designed and conducted to assess the psychometric properties of the P-TICS-M among community-dwelling older adults in Iran.

## METHODS

This is a cross-sectional diagnostic accuracy study that evaluated the sensitivity, specificity, repeatability, and internal consistency of the P-TICS-M instrument in a sample of Iranian older people between 2016 and 2018. The study population consisted of community-dwelling older individuals aged ≥60 years residing in Tehran who were native Persian speakers.

A stratified convenient sampling approach based on cognitive status was utilized as the sampling method for this study. Participants were selected from memory clinics, municipality aged clubs, and retired clubs. The inclusion criteria required participants to be aged ≥60 years, have a telephone, and be native Persian speakers. The exclusion criteria encompassed individuals with severe dementia (global deterioration scale [GDS] ≥6), inability to communicate, with a history of severe psychiatric disorders such as major depression, schizophrenia, opium addiction, or moderate to severe hearing loss.

### Translation and back-translation process

The translation and back-translation process for the TICS-M instrument involved some steps. First, after purchasing the TICS-M instrument and its protocol from the PAR Company, permission was obtained from the developer to translate the tool into the Persian language. The translation followed the WHO protocol and was carried out using the forward-backward method^14^. Two Persian-native experts in the English language translated the TICS-M into Persian. A back-translation was then conducted by another expert who was a native English speaker fluent in Persian. The final back-translated version of the TICS-M was subsequently reviewed and approved by the developer.

### Data gathering

Data gathering in this study involved several tools and assessments. Demographic characteristics were collected through face-to-face interviews, including sex, age, educational level, marital status, and living arrangement, using a questionnaire approved by two experts.

### Data gathering tools

Mood status was evaluated using the Patient Health Questionnaire-9 (PHQ-9), a self-reported diagnostic tool developed by Spitzer et al. It consists of nine items and yields a total score between 0 and 27, with higher scores indicating a greater level of depression. The PHQ-9 is commonly applied to assess depression in older adults^15^. Cognitive abilities were assessed using three instruments. The Abbreviated Mental Test Score (AMTS) was applied, consisting of ten items with the same scoring system for each item (one score for a correct response and zero for an incorrect response). Bakhtiyari et al. evaluated the psychometric properties of this tool in Iran, and a cutoff point of 7 was established to differentiate between individuals with normal and impaired cognition. The results of the study suggested that the abbreviated cognitive test (AMT) was suitable for distinguishing individuals with and without cognitive impairment. The sensitivity and specificity of the AMT at a cutoff point of 8 were 92.15 and 81.5% respectively, based on the comprehensive wasting scale. Using the Diagnostic and Statistical Manual of Mental Disorders (DSM) IV criteria, the sensitivity and specificity of the test were 64.9 and 64.0% respectively, with a cutoff point of 7. The internal reliability of the Persianized AMT was found to be acceptable, with a Cronbach's alpha coefficient of 0.76. This suggests that the test consistently measured cognitive abilities in a reliable manner. The external reliability of the test, when administered by an examiner, was found to be good with an intraclass correlation coefficient of 0.89. This means that different examiners administering the test obtained consistent results, indicating good interrater reliability^16^. The Mini-Mental State Examination (MMSE), a tool that consists of 11 items with a total score of 30 points, was administered to evaluate cognitive functions. A score of 30 represents the best cognitive state, while a score of zero indicates the worst cognitive state. The Persian version of this tool has been validated and shown to have sufficient psychometric properties in Seyedian's study. The Cronbach's alpha coefficient for the whole test was 0.81. Using the receiver operating characteristic (ROC) curve, a score of 22 was considered as the cutoff point, and the test had a sensitivity of 90% and a specificity of 93.5% in this score^17^. The GDS was employed to categorize participants into three groups based on cognitive status: normal cognition (GDS≤2), mild cognitive impairment (GDS=3), and dementia (GDS≥4). This scale, developed by Ruberg et al., ranks individuals according to their cognitive abilities and has been used in multiple validation studies in Iran^18^. The reliability of the PHQ-9 was estimated using Cronbach's alpha coefficient and the multidimensional Rasch model reliability coefficient, which in both cases indicated the optimal validity of this questionnaire^19^.

The TICS-M is an instrument introduced in 1993 that has demonstrated good reliability and validity in various languages for assessing cognitive status among older individuals via telephone^12^. It comprises 13 items and six domains, including orientation, registration and free recall, attention and calculation, comprehension and semantic recent memory, language and repetition, and delayed recall. The TICS-M yields a score ranging from 0 to 50, with a reported sensitivity of 94% and a specificity of 100% for detecting normal cognitive subjects versus those with dementia^20^.

### Assessment of validity

During the qualitative stage of face validity, the scale was administered to ten older adults, and interviews were conducted to assess their perspectives on appropriateness, difficulty, relevancy, and ambiguity. The content validity of the P-TICS-M tool was evaluated by an expert panel consisting of a psychiatrist, a psychologist, a gerontologist, a nurse, and an epidemiologist. For this purpose, we utilized the item content validity index (I-CVI) for each item, which was determined based on the experts’ ratings of relevance using a 4-point ordinal scale. The overall score of scale content validity index (S-CVI) was also calculated, where a score of 1 indicated not relevant, 2 denoted somewhat relevant, 3 represented quite relevant, and 4 signified highly relevant. Furthermore, for each item, the I-CVI was calculated by determining the number of experts who assigned a rating of either 3 or 4 and dividing it by the total number of experts (five). Finally, the I-CVI was calculated as the proportion of items that experts rated as either a score of 3 or 4 in relation to all the items.

The concurrent validity of the P-TICS-M was assessed by calculating the Spearman's correlation coefficient between the scores of the P-TICS-M and the MMSE, GDS, and AMT scores. A correlation coefficient (CC) of 0.7 or greater was deemed to indicate sufficient concurrent validity^21^. The construct validity of the P-TICS-M was evaluated through exploratory factor analysis (EFA) with rotation using Varimax Kaiser Normalization.

### Assessment of reliability

A total Cronbach's alpha coefficient greater than 0.7 was found, confirming the internal consistency of the P-TICS-M^21^, which is considered good. Test-retest reliability was assessed by the same assessor, and the agreement between the total scores of P-TICS-M evaluations conducted twice, with a one-week interval, was determined using the intraclass correlation coefficient (ICC). The ICC of agreement was found to be greater than 0.8, signifying good test-retest reliability^22^.

### Statistical analyses

Analyses were conducted using Statistical Package for Social Sciences (SPSS)^23^ version 21, and Stata^23^ version 12. The EFA was employed to identify the domains of the P-TICS-M. We reported the Kaiser-Meyer-Olkin measure of sampling adequacy and the Bartlett's test of sphericity χ^2^. The ROC analysis was utilized to determine the P-TICS-M cutoff for the screening of dementia, with reference to the categorized AMT, MMSE, and GDS.

### Ethical considerations

This study was approved by the ethical research committee of Tehran University of Medical Sciences (TUMS) under code: IR.TUMS.FNM.REC.1395.267. All the participants as well as a close family member or the legal representative of the participants with dementia signed informed consent.

The present study was conducted as part of a student's thesis for the purpose of obtaining a master's degree. It adhered to all relevant ethical codes and was guided by supervisors who provided ethical guidance throughout the research process. The older adults participants willingly engaged by completing the assessment tools. They had the right to withdraw at any point if they chose to do so. No interventions were performed on the older adults participants. Cognitive examinations were carried out, and if any issues or concerns were identified, the supervisor or the older adults themselves were notified and appropriate actions were taken.

## RESULTS

In this study, a total of 150 older adults aged 60 and above were included. The participants had a mean age of 68.6 years, and 83 (55.3%) of them were female (Table 1). The difference in TICS-M score among cognitive impairment categories is shown in Figure 2. The P-TICS-M score was related to gender and education level, but not to age (Supplementary Material 1 and 2).

**Table 1:** General characteristics of the participants.

Variable	Total population n=150	Normal cognition n=87	MCI n=40	Dementia n=23
Mean age – year (SD)	68.61 (7.41)	66.51 (6.22)	70.30 (7.86)	73.61 (7.92)
Female (%)	83 (55.3)	49 (56.3)	23 (57.5)	11 (47.8)
Mean years of schooling (SD)	8.91 (6.08)	10.20 (5.91)	9.28 (5.56)	3.43 (4.58)
Diabetes (%)	33 (22.0)	17 (19.5)	10 (25.0)	6 (26.1)
Hypertension (%)	62 (41.3)	30 (34.5)	19 (47.5)	13 (56.6)
Cardiovascular disease (%)	29 (19.3)	13 (14.9)	6 (15.0)	10 (43.6)
Heart failure (%)	15 (10.0)	6 (6.9)	7 (17.5)	2 (8.7)
Stroke (%)	6 (4.0)	3 (3.4)	1 (2.5)	2 (8.7)

Abbreviations: SD, standard deviation; MCI, mild cognitive impairment.

**Figure 1:**
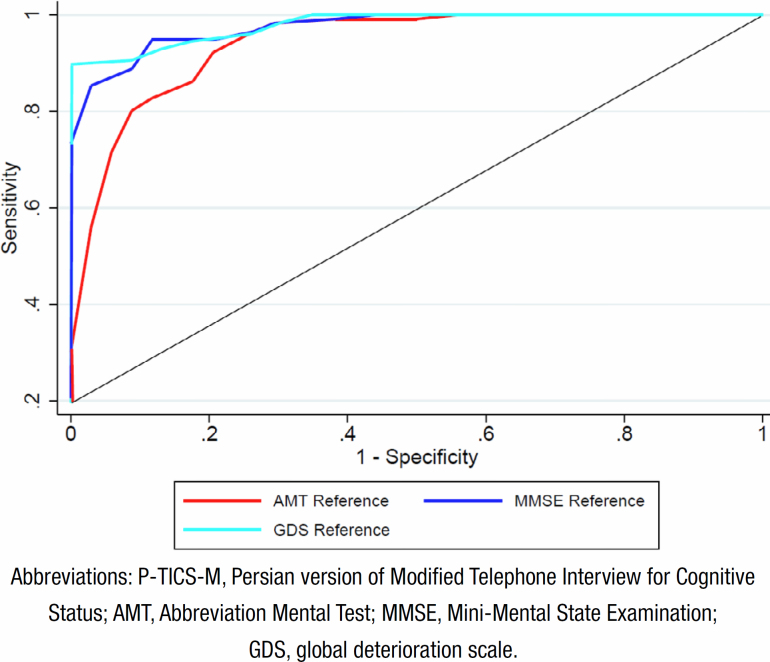
Receiver operating characteristic curve analysis of P-TICS-M score on referencing of AMT, MMSE, and GDS.

**Figure 2:**
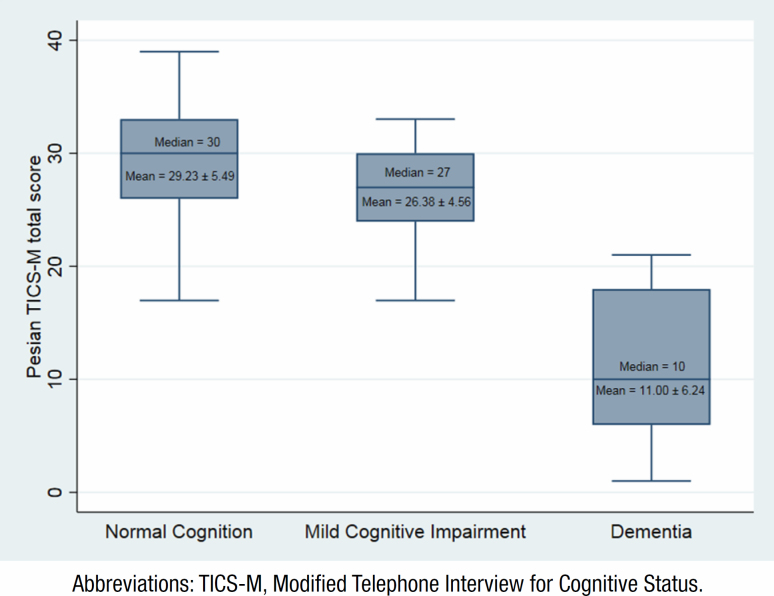
Boxplot TICS-M, and Normal Cognition, Mild Cognitive Impairment, Dementia.

The difference in TICS-M score among cognitive impairment categories is shown in Supplementary Material 2).

The I-CVI for all 13 items of the P-TICS-M was found to be equal to or greater than 0.8, indicating good content validity. The S-CVI for the Persian version of the tool was calculated as 0.892. The Spearman's correlation coefficient can take values from +1 to −1, where +1 indicates a perfect association of ranks, 0 (zero) indicates no association between ranks, and −1 indicates a perfect negative association of ranks. The highest Spearman's correlation coefficient was observed between the MMSE and P-TICS-M scores, with a correlation coefficient (Spearman's Rho) of 0.764. The second-strongest relationship was observed between P-TICS-M scores and AMT (r=0.669). The GDS ranking was correlated with TICS-M scores, with a CC=0.592 (Table 2 and Figure 1). ROC analysis was performed to determine the cutoff point of the P-TICS-M (Figure 1). Based on the referencing of categorized AMT, MMSE, and GDS for the screening of dementia, a cutoff of 22 was identified. The sensitivity for diagnosing dementia using this cutoff was calculated as 92.2, 94.8, and 100%, respectively, while the specificity was 79.4% for AMT≤7, 88.2% for MMSE≤23, and 89.8% for GDS>3 (Table 3). Additionally, a cutoff of >28 was determined using GDS=3 to screen subjects with MCI differing from those with normal cognition. A cutoff of 20 was defined for the differential screening of subjects with dementia from those with MCI (Table 4).

**Table 2:** Correlations between TICS-M scores and other cognitive assessment tools score.

Tool name	Cognitive assessment tools	Spearman's CC	95%CI of CC
TICS-M total scores	MMSE	0.764	0.688–0.823
	AMT	0.669	0.570–0.748
	GDS	0.592	0.477–0.687

Abbreviations: TICS-M, Modified Telephone Interview for Cognitive Status; CC, correlation coefficient; CI, confidence interval.

**Table 3:** Sensitivity and specificity of P-TICS-M on referencing of AMT, MMSE, and GDS cutoff for diagnosis of dementia.

Reference	Cutoff point	Sensitivity (%)	Specificity (%)	LR+	LR-	AUC (95%CI)
AMT>7	22	92.2 (85.4–96.2)	79.4 (61.6–90.7)	4.48	0.10	0.936 (0.880–0.968)
MMSE>22	22	94.8 (88.6–97.9)	88.2 (71.6–96.2)	8.06	0.06	0.974 (0.933–0.993)
GDS>3	22	100 (82.2–100)	89.8 (82.8–94.2)	>10.5	0.10	0.979 (0.943–0.996)

Abbreviations: P-TICS-M, Persian version of Modified Telephone Interview for Cognitive Status; AMT, Abbreviated Mental Test; MMSE, Mini-Mental State Examination; GDS, global deterioration scale; LR+, positive likelihood ratio; LR-, negative likelihood ratio; AUC, area under the receiver operating characteristic (ROC) curve; CI, confidence interval.

**Table 4:** The sensitivity and specificity P-TICS-M for distinguishing normal cognitive subjects from those with MCI and subjects with MCI from those with dementia on criterion of GDS.

Diagnosis	P-TICS-M tool			
	Cutoff point based on GDS	Specificity (%)	Sensitivity (5)	AUC (95%CI)
MCI from normal cognition	28	66.7	55	0.661 (0.572–0.743)
Dementia from MCI	20	87	90	0.972 (0.900–0.996)

Abbreviations: P-TICS-M, Persian version of Modified Telephone Interview for Cognitive Status; MCI, mild cognitive impairment; GDS, global deterioration scale; CI, confidence interval; AUC, area under the receiver operating characteristic (ROC) curve.

We used EFA for detecting the domains of P-TICS-M and then compared these with the domains that the developer defined for this tool. In EFA four domains were explored. Domain one included orientation, knowing the phone number, counting backward from 20 to 1, taking away seven from 100, opposite of darkness, repeating the word, who is the president, and who is the leader. The second domain consisted of registration and recall. The third domain had two items: an Iranian famous fragrant flower and knowing his or her age, and the fourth domain contained one item: the instrument used for cutting paper. The Kaiser-Meyer-Olkin measure of sampling adequacy was 0.849, Bartlett's test of sphericity was 957.79, and p<0.05 (Table 5).

**Table 5:** Exploratory factor analysis with rotation Varimax Kaiser Normalization*.

Exploratory factor analysis: Rotated Component Matrixᵃ					
Items		Correlation Matrix			
		Factor 1	Factor 2	Factor 3	Factor 4
Factor 1	Orientation	**0.814**	0.052	0.268	-0.052
	Know phone number	**0.807**	0.182	0.073	-0.172
	Count backward from 20 to 1	**0.783**	0.012	0.225	0.002
	Subtract 7 from 100	**0.766**	0.017	0.254	0.024
	Opposite of darkness	**0.711**	0.161	-0.315	0.221
	Repeat the word	**0.697**	0.229	-0.249	0.320
Factor 2	Who is the president?	**0.694**	0.034	0.297	-0.129
	Who is the leader?	**0.529**	-0.088	0.197	0.298
Factor 3	Registration	0.066	**0.958**	0.088	0.034
	Recall	0.105	**0.953**	0.090	0.006
Factor 4	Iranian fragrant flower	0.133	0.158	**0.659**	0.339
	Know her or his age	0.396	0.097	**0.569**	-0.206
	Instrument used to cut paper	-0.028	0.024	0.053	**0.873**

Notes:

* Kaiser-Meyer-Olkin measure of sampling adequacy, 0.707; χ^2^=, 902.220; Df (Degrees of Freedom), 78.

The internal consistency of the P-TICS-M instrument was assessed using Cronbach's alpha. The coefficient for all 39 items of the P-TICS-M was found to be 0.920, indicating excellent internal consistency. The Cronbach's alpha coefficient for the seven domains of the P-TICS-M was calculated as 0.857, indicating good internal consistency at the domain level (Table 6). Test-retest reliability was evaluated by repeating the measurements of the instrument by the same rater after a one-week interval for a randomly selected group of 15 participants. The absolute agreement between the test and retest scores for all domains of the P-TICS-M was higher than 0.9, suggesting excellent test-retest reliability (Table 6).

**Table 6:** Interclass correlation (absolute agreement) between score of domains also total score of Iranian versions of TICS-M in intra-rater test-retest.

Items	ICC	95%CI
Orientation score	0.932	0.796–0.977
Semantic memory score	1.000	-
Registration	0.941	0.824–0.980
Attention	0.952	0.858–0.984
Language	0.939	0.831–0.979
Recall	0.936	0.781–0.979
Total TICS-M score	0.947	0.845–0.982

Abbreviations: TICS-M, Modified Telephone Interview for Cognitive Status; ICC, intraclass correlation coefficient; CI, confidence interval.

## DISCUSSION

In this cross-sectional study, our aim was to assess the validity and reliability of the Iranian version of the TICS-M in a sample of community-dwelling older adults residing in Tehran. The TICS-M is recognized as one of the most widely used telephone-based instruments for evaluating cognitive function worldwide. The use of telephone-based instruments for this purpose can significantly expand the reach of screening programs designed to identify cognitive impairment in the community. This aligns with the objectives outlined in the WHO's action plan for dementia, which emphasizes the need for comprehensive screening programs to address cognitive impairment at a population level^24^. To ensure the accuracy and comprehensibility of the P-TICS-M, a standardized translation process was followed based on the International Physical Activity Questionnaire (IPAQ) protocol for translation^25^.

Face validity of the P-TICS-M was assessed through interviews with ten healthy older adults. Their feedback and input were considered to make necessary revisions to the original TICS-M, resulting in the development of the P-TICS-M. One specific modification was made by changing the name of a flower from “prickly green plant” to “Mohammadi flower” as the latter is a more familiar term to the older adults population. Another adjustment made was replacing the term “Methodist Episcopal” with the name “Malekoshoaraye Bahar”, which was confirmed by linguistic experts to have the same difficulty in pronouncing words.

The content validity of the P-TICS-M was established by consulting experts proficient in English. They confirmed that the domains of the P-TICS-M were similar to those in the original English version of the tool and covered the majority of cognitive functions. These steps ensured the face and content validity of the P-TICS-M and ensured that it was appropriate for use in the Iranian population.

We observed a CC>0.7 between P-TICS-M score and MMSE, indicating a significant concurrent validity. Similar findings have been reported in several studies conducted in different languages, supporting the good concurrent validity of TICS-M. For instance, a study conducted in Israel reported a CC=0.82 between TICS-M and MMSE^26,27^. Similarly, a study involving women in France found a CC=0.72 between TICS-M and MMSE scores^28^. A Korean study by Seo et al. reported a CC=0.75 between the Korean version of TICS-M and MMSE. Furthermore, they observed a CC=0.63 between P-TICS-M scores and the clinical dementia rating scale^29^.

The construct validity of TICS-M was assessed through EFA. The identified components were in line with the six domains defined by the developer. Notably, registration and recall were grouped together in a single component, which is conceptually logical (data not shown). Despite the slight deviation from the developer's predefined domains, the grouping of items in P-TICS-M remained largely similar^26,30^.

Regarding internal consistency, P-TICS-M demonstrated excellent reliability, as indicated by Cronbach's alpha>0.90^31^. Additionally, P-TICS-M exhibited good test-retest reliability, with an ICC>0.9 between two sets of scores obtained one week apart. This finding suggests excellent agreement between the two measurements. Our reliability results for P-TICS-M align with findings from other studies conducted in various communities^32,33^.

In our study, a cutoff level of 22 for P-TICS-M yielded a sensitivity >90% and the specificity was almost ≥80% for distinguishing individuals with dementia and those with normal cognition. These results indicate the high accuracy of P-TICS-M in screening for dementia. These high sensitivity and specificity values make P-TICS-M suitable for both research and clinical applications in Iran. It's worth noting that while the sensitivity and specificity slightly decreased in illiterate participants, an area under the ROC curve (AUC) >90% in the illiterate group still demonstrates a high diagnostic accuracy (data not shown). Furthermore, our study found a high positive likelihood ratio (LR+) and a very low negative likelihood ratio (LR-) for P-TICS-M, further supporting its utility for screening individuals with dementia in clinical practice. These findings align with other studies evaluating the psychometric properties of TICS-M in different languages. For example, Barber et al. reported an AUC of 0.94 and a cutoff score of 20, which produced a sensitivity of 0.92 and specificity of 0.80 for detecting dementia in post-stroke subjects^4^. Another study reported a sensitivity of 83% and specificity of 100% for the TICS-M to differentiate subjects with dementia from those with normal cognition^30,34^. According to the AUC for MCI screening, it seems that the Persian version of this tool does not have an excellent performance for distinguishing subjects with MCI from those who have normal cognition status, and also has a limited ability to distinguish MCI from dementia. Then it seems that P-TICS-M has relatively low accuracy in differentiating individuals with MCI from those with neurocognitive disorders. Marsiske et al. reported a high accuracy of TICS-M for screening MCI in the United States^20^. However, some other studies reported that the TICS-M is an effective instrument for screening MCI among community-dwelling older adults^10,34^. This discrepancy in results may be attributed to the fact that while the GDS criterion is highly applicable for diagnosing dementia, it may not be a very accurate tool for detecting MCI when compared to dementia. Additionally, the reported prevalence of MCI varied significantly across studies, likely due to differences in the definition of MCI and the need for a culturally adapted definition of this condition^34^.

To the best of our knowledge, this is the first study in Persian-speaking countries to evaluate the properties of TICS-M. We employed several tools to assess the validity of P-TICS-M, confirming its high validity compared to similar tools. This instrument can be valuable in research and clinical screening projects, contributing to the cost reduction of community-based surveys on the cognitive status of older populations.

It is important to note some limitations of our study. We were unable to use clinician screening criteria as gold standard tools for the validation of P-TICS-M. Therefore, we suggest conducting a large-scale study for the general clinical use of P-TICS-M. Additionally, it is recommended to evaluate the validity of P-TICS-M for diagnosing MCI compared to normal cognition, based on the clinical judgment of expert clinicians. Another limitation of our study was the difficulty in accessing older adults with cognitive impairment, resulting in the utilization of convenient sampling.

In conclusion, the P-TICS-M tool appears to be effective in screening cognitive disorders and differentiating between individuals with dementia and those with normal cognition. A strong correlation with face-to-face tools that was found indicated that it may be a convenient alternative that does not require physical presence or extensive training. Compared to other tools like MMSE, the P-TICS-M is less limited in terms of cost, time, and resources needed for screening. It demonstrated better sensitivity in distinguishing individuals with MCI from those with normal cognition. However, it may not be as accurate in detecting MCI among Iranian older adults individuals. Despite this limitation, the P-TICS-M showed high internal and external reliability, making it a suitable tool for evaluating and identifying individuals at dementia risk in both research and clinical settings. Its strong sensitivity and specificity make it valuable for identifying older adults at risk of dementia.
